# The impact of sex and physical performance on long-term mortality in older patients with myocardial infarction

**DOI:** 10.1186/s12916-021-02211-1

**Published:** 2022-01-20

**Authors:** Elisabetta Tonet, Albert Ariza-Solé, Matteo Serenelli, Francesc Formiga, Juan Sanchis, Rita Pavasini, Pablo Diez-Villanueva, Francesco Vitali, Clara Bonanad, Giovanni Grazzi, Antoni Carol, Giorgio Chiaranda, Graziella Pompei, Laura Sofia Cardelli, Serena Caglioni, Federico Gibiino, Stefano Volpato, Gianluca Campo

**Affiliations:** 1 Cardiovascular Institute, Azienda Ospedaliero-Universitaria di Ferrara, Cona, FE Italy; 2 Cardiology Unit, Azienda Ospedaliero Universitaria of Ferrara, 44124 Ferrara, Italy; 3 Cardiology Department, Hospital Universitari de Bellvitge, IDIBELL, L’Hospitalet de Llobregat, Barcelona, Spain; 4 Internal Medicine, Hospital Universitari de Bellvitge, IDIBELL, L’Hospitalet de Llobregat, Barcelona, Spain; 5 Cardiology Department, Hospital Clínico de Valencia, INCLIVA, Universidad de Valencia, CIBER CV, Valencia, Spain; 6 Hospital Universitario La Princesa, Madrid, Spain; 7 Public Health Department and Center of Sport and Exercise Sciences, University of Ferrara, Ferrara, Italy; 8 Servicio de Cardiologia, Hospital Moisès Broggi, Sant Joan Despi, Barcelona, Spain; 9 Department of Public Health, AUSL Piacenza, and Sport Medicine Service, Piacenza, Italy; 10 Department of Medical Science, University of Ferrara, Ferrara, Italy

**Keywords:** Acute coronary syndrome, Sex, Physical performance, Elderly, Mortality

## Abstract

**Background:**

Sex influences outcome of patients with acute coronary syndrome (ACS). If there is a relationship between sex and physical performance is unknown.

**Methods:**

The analysis is based on older (≥70 years) ACS patients included in the FRASER, HULK, and LONGEVO SCA prospective studies. Physical performance was assessed by Short Physical Performance Battery (SPPB). The primary outcome was all-cause mortality.

**Results:**

The study included 1388 patients, and 441 (32%) were women. At presentation, women were older and more compromised than men. After a median follow-up of 998 [730–1168] days, all-cause death occurred in 334 (24.1%) patients. At univariate analysis, female sex was related to increased risk of death. After adjustments for confounding factors, female sex was no longer associated with mortality. Women showed poor physical performance compared with men (*p* < 0.001). SPPB values emerged as an independent predictor of death. Including clinical features and SPPB in the multivariable model, we observed a paradigm shift in the prognostic role of female sex that becomes a protective factor (*HR* 0.73, 95% *CI* 0.56–0.96). Sex and physical performance showed a significant interaction (*p* = 0.03). For lower SPPB values (poor physical performance), sex-related changes in mortality were not recorded, while in patients with higher SPPB values (preserved physical performance), female sex was associated with better survival.

**Conclusions:**

Two key findings emerged from the present real-life cohort of older ACS patients: (i) physical performance strongly influences long-term mortality; (ii) women with preserved physical performance have a better outcome compared to men.

**Trial registration:**

www.clinicaltrials.gov NCT02386124 and NCT03021044

**Supplementary Information:**

The online version contains supplementary material available at 10.1186/s12916-021-02211-1.

## Background

A recent analysis in patients admitted for myocardial infarction at young age (< 50 years) highlighted the poorer prognosis of women when compared to men [1]. Over the past few years, several studies investigated the intricate relationship between sex and long-term outcome after acute coronary syndrome (ACS) [2–5]. Overall, data show that compared to men, at hospitalization, women are older and present a higher risk profile because of the heavy burden of comorbidities [5]. After adjusting for potential confounding factors, female sex emerged as an independent predictor of poor outcome [5]. The common drawback of these studies is the difficulty to adjust for all potential confounding factors. Indeed, while in younger ACS patients, the discrepancies are mainly related to a different distribution of risk factors and comorbidities, in the older ones, the differences could be linked to important, but unrecognized prognostic factors, such as malnutrition, frailty, and physical performance [6]. Indeed, it is well established that chronological and biological age may differ considerably in older ACS patients, and the prognosis is mainly guided by the second one [7, 8]. Therefore, it is not surprising that tools for assessing physical performance, such as the Short Physical Performance Battery (SPPB), were demonstrated to be good indicators of health status in older adults [8]. Our analysis aims to analyze the effect of sex and SPPB on outcome. In particular, the present study sought to evaluate if the adverse prognostic role of female sex is confirmed in a population of older (≥70 years) patients with ACS and how sex and physical performance interact to affect the outcome.

## Methods

### Study design

The present study is not a prespecified post hoc analysis carried out by using data of the populations from three different studies. The first one was the “Frailty in Elderly Patients Receiving Cardiac Interventional Procedures” (FRASER) study (NCT02386124) [8, 9]. The FRASER is a multicenter observational prospective study involving four Italian hospitals and analyzing the frailty status of 402 adults aged ≥70 years with ACS diagnosis demonstrating that SPPB had the greatest incremental value in outcome prediction model [8, 9]. The second study was the “Physical Activity Intervention for Elderly Patients with Reduced Physical Performance after ACS” (HULK) study, a prospective, multicenter randomized clinical trial, which screened the physical performance of 487 ACS patients aged 70 years old and over undergoing PCI because of ACS (NCT03021044). The study’s primary aim was to assess the effect of a tailored exercise intervention on physical performance demonstrating its benefit at 6- and 12-month follow-up [10–12]. The third study was the “Impacto de la Fragilidad y otros Síndromes Geriátricos en el Manejo y Pronóstico Vital del Anciano con Síndrome Coronario Agudo sin Elevación de Segmento ST” (LONGEVO SCA) registry. This was a multicenter registry conducted to assess the characteristics of 532 adults aged ≥80 years admitted to 44 Spanish hospitals with a diagnosis of non-ST segment elevation ACS in order to evaluate the effect of frailty on 6-month morality and mortality and readmission [13, 14]. In all the cohorts, physical performance was prospectively assessed by the SPPB. The studies were conducted following the ethical principles of the Declaration of Helsinki. All patients were informed that their participation was voluntary, and all of them gave written informed consent. The ethical review boards of the participating hospitals approved the studies.

### Study measurements

A large amount of common clinical data, including demographics, previous medical history, comorbidities, laboratory data, and treatments, was collected in the three studies. Data were collected using the same method across studies. Starting from each study’s individual dataset, a single database was generated under the principal investigators’ supervision (ET, GC, and AAS).

### Short Physical Performance Battery (SPPB)

The SPPB scale was prospectively collected after the acute phase (after mobilization), but before hospital discharge. In all three studies [8, 10, 13], the SPPB scale is an easy-to-perform battery consisting of three different sections designed to assess lower limb function. The SPPB score ranges from 0 (worst performance) to 12 (best performance) [15, 16]. Previous studies and meta-analysis showed that SPPB value is strongly related to all-cause mortality and the changes of one single unit are clinically meaningful [9, 17, 18]. The prognostic role of SPPB was independent of underlying disease (cardiac or not) and setting (in-hospital vs. outpatient) [17, 18].

### Outcomes

The primary outcome of the present analysis was all-cause mortality. Each study included a follow-up with regular outpatient visits. Clinical status, medical treatment and compliance, laboratory data, and adverse events were recorded in each visit. The median follow-up of the study population of the present analysis was 998 [730–1168] days. In case of an adverse event, relevant data and source documentation were collected. All events were adjudicated by a clinical events committee whose members were unaware of the patients’ baseline characteristics. Death was collected consulting in-hospital registries, direct contact with patients’ relatives, and consulting regional mortality registries.

### Statistical analysis

Continuous data were tested for normal distribution with the Kolmogorov–Smirnov test. Normally distributed values were presented as mean ± SD and compared by *T*-test and one-way analysis of variance; otherwise, median value (interquartile range [IQR]), the Mann–Whitney *U* test, and the Kruskal–Wallis test were used. Categorical variables were summarized in terms of counts and percentages and compared by using the two-sided Pearson’s chi-squared test. Association of baseline variables with outcome was tested using Cox regression. The proportional hazards assumption was examined graphically and with the use of the Schoenfeld residuals. A directed acyclic graph (DAG) was drawn to represent the relationship between the exposure (sex) and the outcome, also showing inter-relationships between the various covariates, exposure, and outcome (see Additional file 1: Fig. S1). This method was used to choose the best adjustment set to design the multivariate Cox regression models. To permit comparison with previous studies, a first model including only clinical and laboratory variables was generated (multivariable model 1). After that, a second multivariable model including SPPB value was implemented (multivariable model 2). The effect of sex on outcome according to SPPB was further explored using a cubic spline method modeling SPPB as a continuous variable and showing how the effect of sex on outcome change with incremental values of SPPB. Additionally, considering that the HULK study was a randomized trial and that it provided a physical activity intervention in a part of patients, we performed two other analyses: an individual participant-data metanalysis and a multivariate analysis including physical activity program as an adjustment term.

The cumulative risk of primary outcome according to gender was graphically reported using the Kaplan–Meier method, and groups (men vs. women) were compared with the log-rank test. The best cut-off for prediction of all-cause mortality by SPPB was identified applying the concordance probability method of Liu [19]. In addition, to provide a graphical representation of the relationship of gender with outcome, we reported adjusted survival curves. Survival curves were adjusted by the covariates included in the multivariate model. In more details, we adjusted the survival function to the overall means of age, ejection fraction, creatinine clearance, hemoglobin, and white blood cell, while also adjusting the estimates to the base levels of the factor variables Killip status, previous myocardial infarction, revascularization, peripheral artery disease, and diabetes. The statistical significance was defined as *p* < 0.05. All analyses were performed with Stata 16.

## Results

### Study population

Starting from 1419 ACS patients, we excluded 31 (2.2%) patients due to missing data. Thus, the final study population involved 1388 older ACS patients (*n* = 402 subjects from the FRASER study, *n* = 485 from the HULK study, and *n* = 501 from the LONGEVO-SCA registry). The mean age was 81 [75–84] years, and 441 (32%) were women. Table 1 shows baseline characteristics of the entire study population and after stratification according to sex. Women showed worse clinical presentation with a higher prevalence of hypertension, a worse Killip class, lower values of hemoglobin and creatinine clearance, and higher values of low-density lipoprotein. Invasive strategy was less performed in women (87% vs. 95%, *p* < 0.001).

**Table 1:** Baseline characteristics

	Total (***n*** = 1388)	Male (***n*** = 947)	Female (***n*** = 441)	***p***
Age (years)	81 [75–84]	80 [75–83]	82 [77–85]	< 0.001
BMI (kg/m^2^)	26.4 [24.1–29]	26.3 [24.3–28.7]	26.4 [23.5–29.4]	0.34
**CV risk factors, no. (%)**				
Diabetes	465 (33.5)	323 (34.1)	142 (32.2)	0.47
Hypertension	1170 (84.4)	782 (82.7)	388 (88.1)	0.011
Hyperlipidemia	775 (55.9)	536 (56.7)	239 (54.2)	0.39
Current smoker	387 (30.1)	335 (35.4)	52 (11.8)	< 0.001
**Medical history, no. (%)**				
MI	445 (32.1)	351 (37.1)	94 (21.3)	< 0.001
PCI	397 (28.6)	300 (31.7)	97 (22)	< 0.001
CABG	146 (10.5)	132 (14)	14 (3.2)	< 0.001
PAD	286 (20.6)	221 (23.4)	65 (14.7)	< 0.001
**Clinical presentation**				
STEMI (%)	284 (20.5)	185 (19.5)	99 (22.4)	0.21
Killip class > I, no. (%)	232 (16.8)	137 (14.6)	95 (21.7)	0.001
**Laboratory data (at inclusion)**				
White blood cells, (u/μl)	7.8 [6.6–9.6]	6.7 [5.7–7.8]	6.6 [5.5–7.8]	0.58
Hemoglobin, (g/dl)	12.7 [11.3–14]	13.1 [11.6–14.3]	12 [10.7–13]	< 0.001
Creatinine clearance, (ml/min)	53 [39–67.3]	55 [41.4–68.3]	47.5 [34.5–61.3]	< 0.001
Low-density lipoprotein (mg/dl)	93.2 [68.6–125]	90 [66–119]	107.6 [76-137]	< 0.001
**Other data**				
LVEF (%)	54 [44–60]	54 [44–60]	54 [44–60]	0.35
Invasive strategy, no (%)	1283 (92.4)	899 (95.1)	384 (86.4)	< 0.001
Multivessel disease, no (%)	880 (70.1)	645 (73.4)	235 (62)	< 0.001
Coronary revascularization, no (%)	1238 (89.2)	870 (92)	368 (83.4)	< 0.001

*BMI* body mass index, *MI* myocardial infarction, *PCI* percutaneous coronary intervention, *CABG* coronary artery bypass graft, *PAD* peripheral artery disease, *STEMI* ST segment elevation myocardial infarction, *NSTEMI* non-ST segment elevation myocardial infarction, *UA* unstable angina, *LVEF* left ventricular ejection fraction

### All-cause mortality

**Fig. 1:**
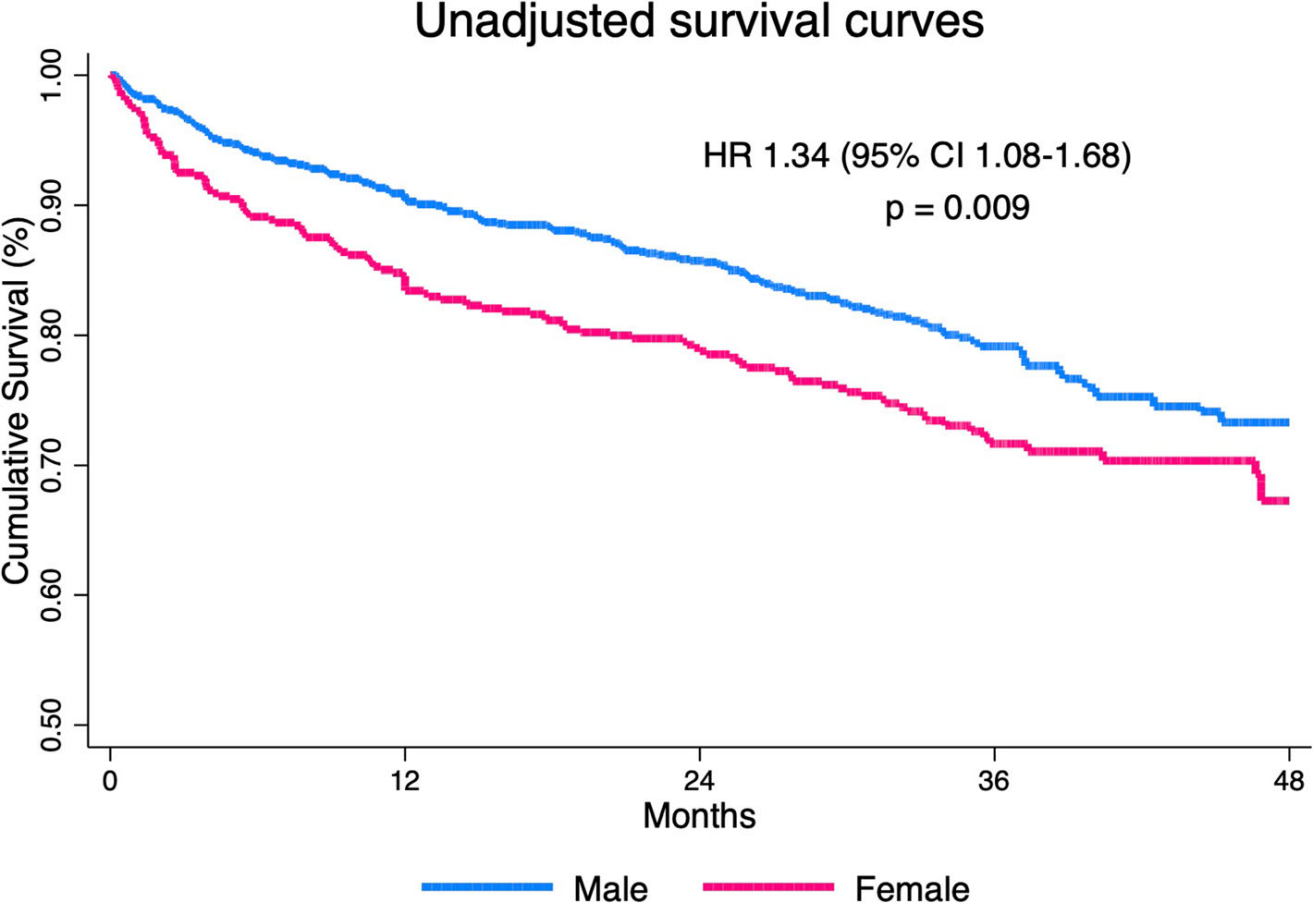
Unadjusted survival curve for all-cause mortality according to sex

Overall, all-cause mortality occurred in 334 (24.1%) patients. More specifically, it occurred in 125 (28.3%) women and 209 (22.1%) men (*p* = 0.011). Unadjusted survival curves after stratification for sex are shown in Fig. 1. At the univariate analysis, age, female sex, prior myocardial infarction (MI), peripheral artery disease, Killip class, white blood cells, hemoglobin, creatinine clearance, and ejection fraction were associated with all-cause mortality (Table 2). At multivariable analysis, including clinical and laboratory variables (model 1 in Table 2), age, peripheral artery disease, Killip class, hemoglobin, creatinine clearance, ejection fraction, white blood cell, and revascularization emerged as independent predictors of all-cause mortality (Table 2). In this model, female sex was not independently associated with all-cause mortality. Adjusted survival curves confirmed a similar outcome between men and women (*p* = 0.85) (Additional file 2: Fig. S2).

**Table 2:** Univariate and multivariate analyses for all-cause mortality

	Univariate		Multivariable model 1*		Multivariable model 2†	
	***HR*** (95% ***CI***)	***p***	***HR*** (95% ***CI***)	***p***	***HR*** (95% ***CI***)	***p***
Age (years)	1.13 (1.11–1.15)	< 0.001	1.07 (1.04–1.10)	< 0.001	1.05 (1.02–1.08)	< 0.001
Female sex (%)	1.34 (1.076–1.67)	0.009	0.98 (0.75–1.26)	0.85	0.75 (0.57–0.99)	0.042
**CV risk factors, no. (%)**						
Diabetes	1.13 (0.9–1.41)	0.28	0.96 (0.74–1.25)	0.77	0.82 (0.62–1.10)	0.19
**Medical history, no. (%)**						
MI	1.41 (1.13–1.75)	0.002	1.07 (0.83–1.38)	0.61	1.07 (0.82–1.38)	0.63
PAD	1.33 (1.04–1.7)	0.022	1.31 (0.99–1.73)	0.062	1.22 (0.91–1.63)	0.18
**Clinical presentation**						
Killip class > I, no. (%)	3.46 (2.75–4.34)	< 0.001	1.79 (1.37–2.35)	< 0.001	1.36 (1.02–1.81)	0.038
**Laboratory data (at inclusion)**						
White blood cells, (u/μl)	1.08 (1.05–1.11)	< 0.001	1.04 (1.00–1.07)	0.050	1.03 (1.00–1.07)	0.084
Hemoglobin, (g/dl)	0.84 (0.8–0.9)	< 0.001	0.92 (0.87–0.98)	0.011	0.93 (0.87–0.99)	0.030
Creatinine clearance, (ml/min)	0.97 (0.96–0.97)	< 0.001	0.99 (0.98–1.00)	0.002	0.99 (0.98–0.99)	< 0.001
**Other data**						
LVEF (%)	0.98 (0.96–0.98)	< 0.001	0.98 (0.97–0.99)	< 0.001	0.98 (0.97–0.99)	< 0.001
Coronary revascularization	0.3 (0.22–0.38)	< 0.001	0.68 (0.49–0.96)	0.026		
SPPB	0.8 (0.77–0.83)	< 0.001	NC		0.87 (0.84–0.91)	< 0.001

*Model 1 shows multivariable model for all-cause death including clinical and laboratory variables (except for SPPB). C-statistic = 0.74. NC not considered in the present multivariable model

†Model 2 shows multivariable model including clinical and laboratory variables and SPPB values. C-statistic = 0.76

*BMI* body mass index, *MI* myocardial infarction, *PCI* percutaneous coronary intervention, *CABG* coronary artery bypass graft, *PAD* peripheral artery disease, *STEMI* ST segment elevation myocardial infarction, *LVEF* left ventricular ejection fraction, *SPPB* Short Physical Performance Battery

### Physical performance in women vs. men

**Fig. 2:**
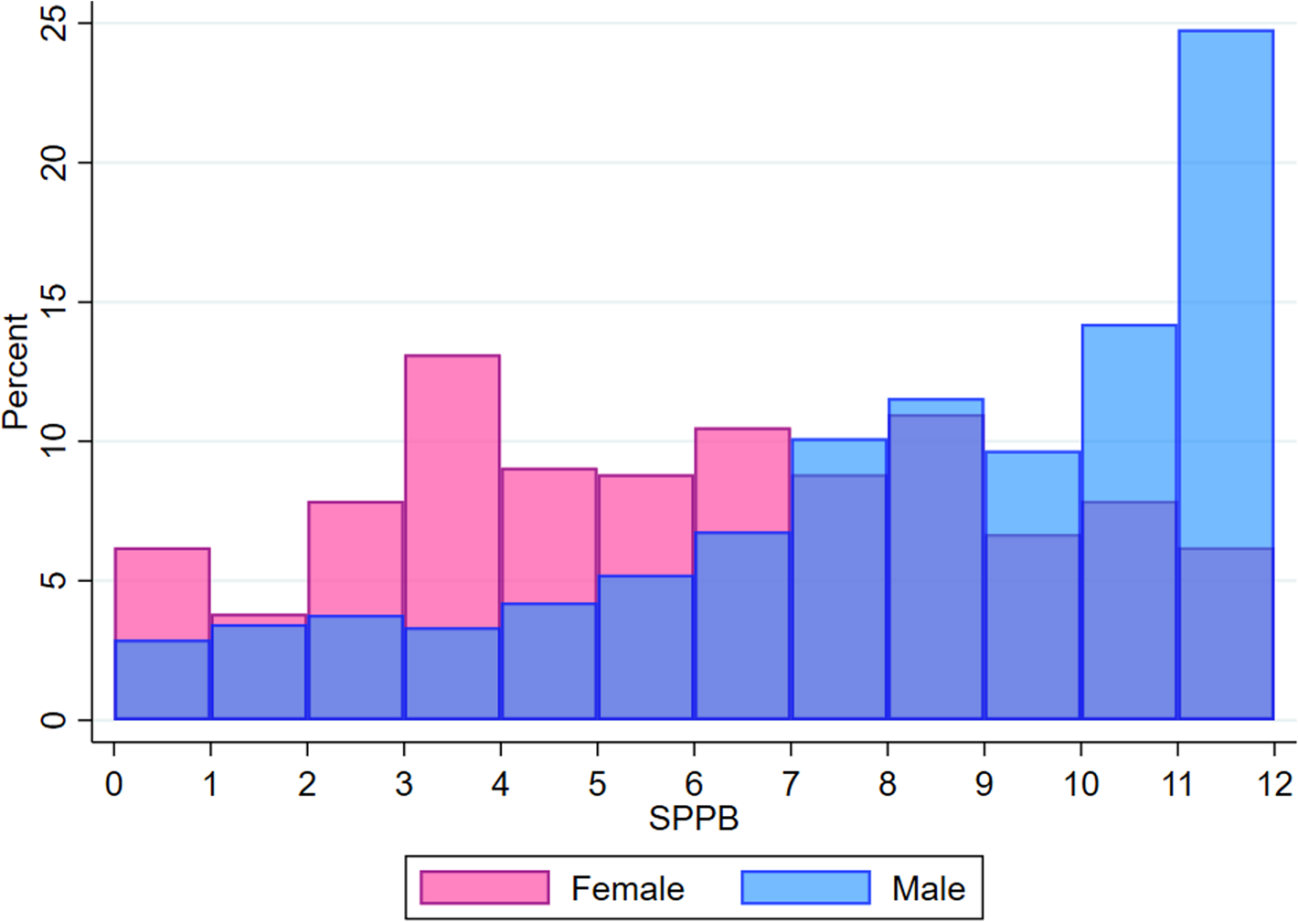
SPPB values in women and men. SPPB Short Physical Performance Battery

Overall, the median SPPB score was 8 [5–10] points. SPPB values in men and women are shown in Fig. 2. SPPB values were significantly lower in women than men (6 [3–8] vs. 8 [6–10] respectively, *p* < 0.001).

### Sex, physical performance, and all-cause mortality

**Fig. 3:**
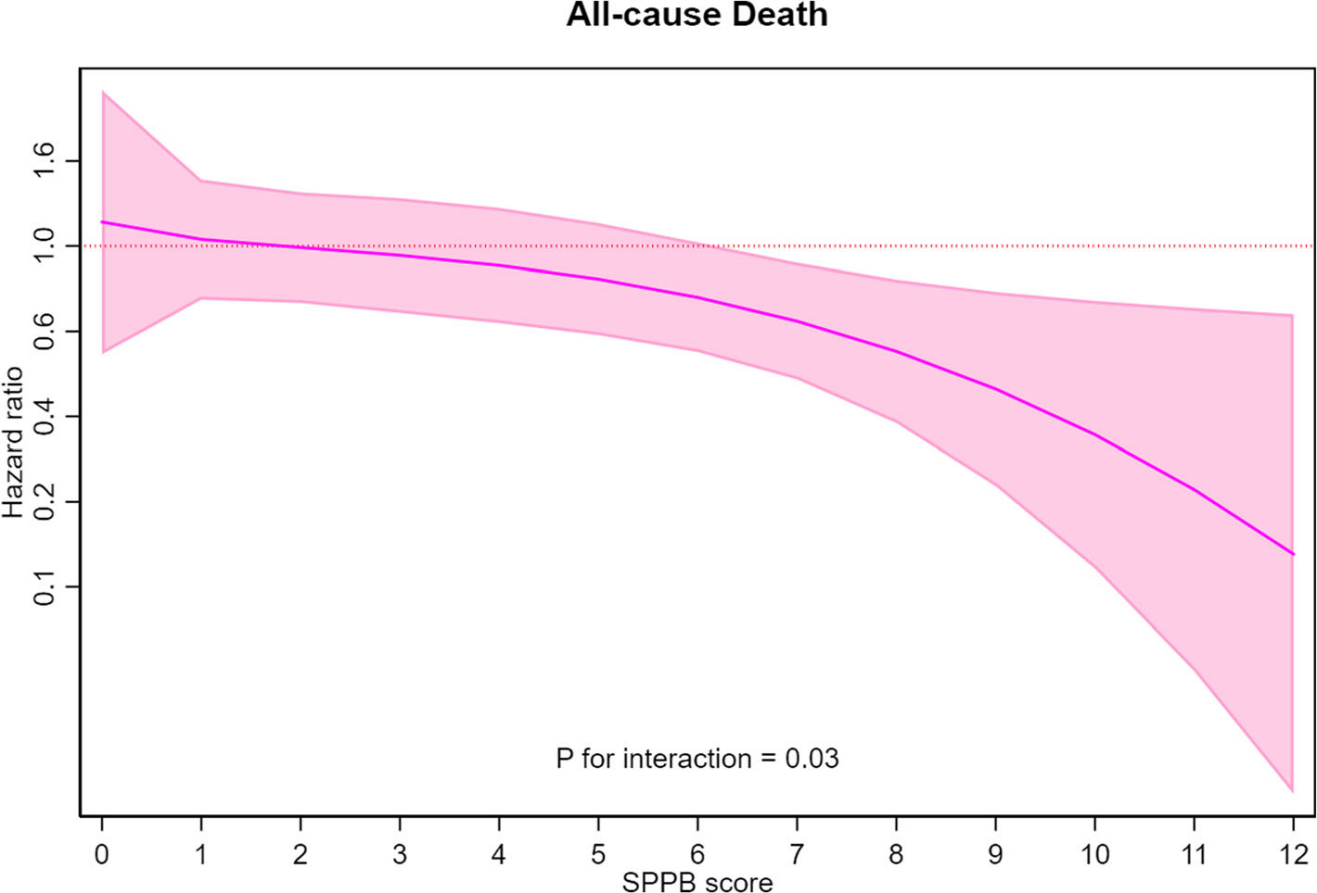
Interaction between sex and physical performance on all-cause mortality. Spline curve showing interaction between SPPB values and female sex on outcome. The curve shows that sex has the strongest predictive value with HR < 1 for the highest SPPB values (*p* for interaction = 0.03)

**Fig. 4:**
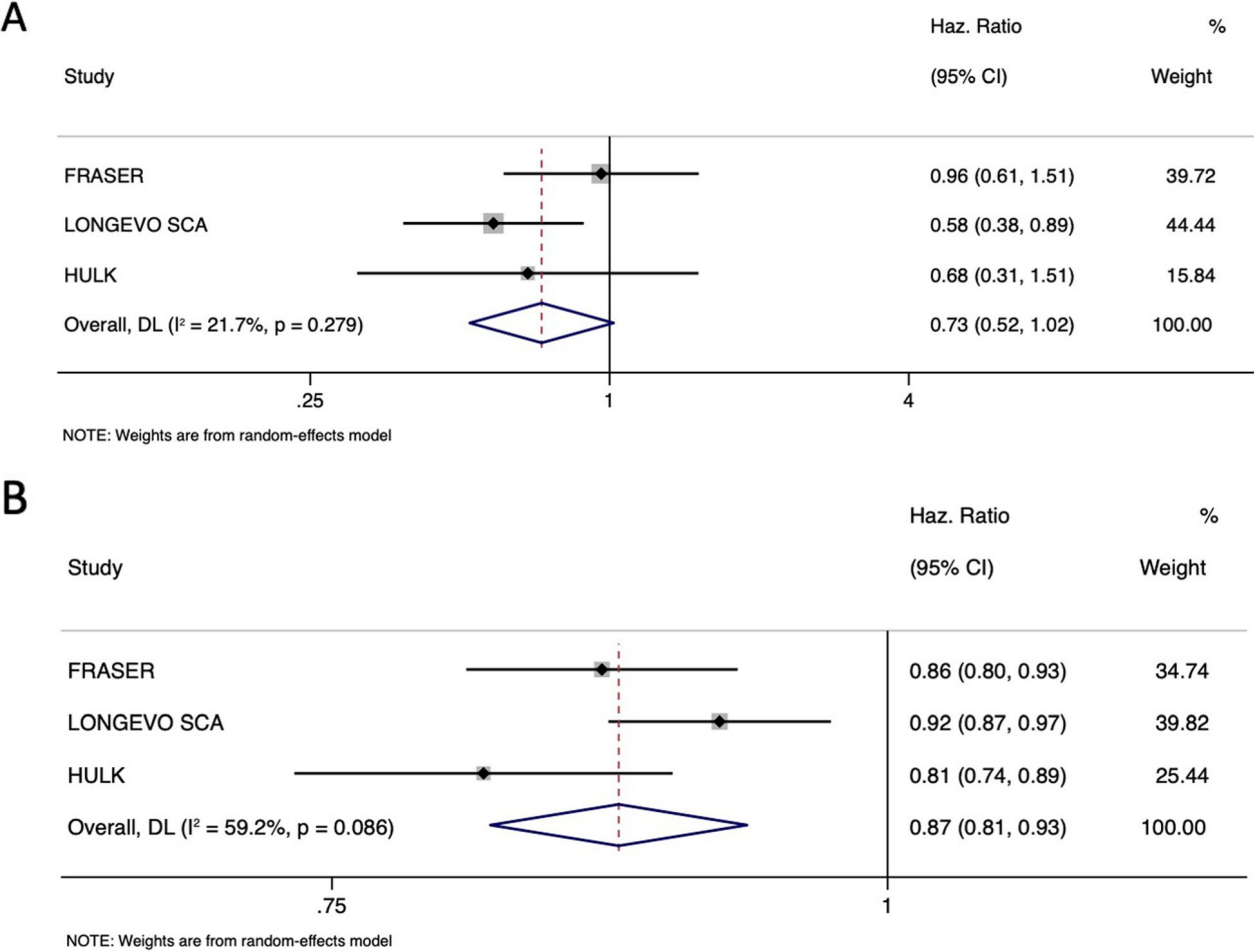
Individual participant-data metanalysis. **A** Sex HR in each study and in overall population, including physical activity program in the multivariate analysis. Plot shows no changes in outcome prediction. **B** SPPB HR in each study and in overall population, including physical activity program in the multivariate analysis. Plot shows no changes in outcome prediction

The univariate analysis revealed a strong predictive value of SPPB (*HR* 0.80, 95% *CI* 0.77–0.83, *p* < 0.001) (Table 2). After the inclusion of SPPB in the multivariable model (model 2 in Table 2), age (*HR* 1.05, 95% *CI* 1.02*–*1.08), previous MI (1.07, 95% *CI* 0.82–1.38), Killip class (*HR* 1.36, 95% *CI* 1.02–1.81), hemoglobin (*HR* 0.93, 95% *CI* 0.87–0.99), creatinine clearance (*HR* 0.99, 95% *CI* 0.98–0.99), left ventricle ejection fraction (*HR* 0.98, 95% *CI* 0.97–0.99), and SPPB (*HR* 0.87, 95% *CI* 0.84–0.91) resulted independent predictors of all-cause mortality. As compared to the multivariable model including only clinical and laboratory features, in the model also considering SPPB, female sex became a protective factor for all-cause mortality (*HR* 0.75, 95% *CI* 0.57–0.99) (Table 2, Additional file 3: Fig. S3). The relationship between sex, SPPB values, and outcome after adjustment for confounding factors is shown in Fig. 3: it can be noted that female sex changed its effect (central line) according to SPPB values, acquiring a stronger protective effect for higher SPPB values. While for lower SPPB values the influence of sex on mortality is not a decisive factor, in patients with higher SPPB values (*p* for interaction = 0.03), female sex was associated with better survival rate. We found 8 as the best cut-off of SPPB for prediction of all-cause mortality and divided patients in two groups. In patients with SPPB value ≥ 8, female sex confirmed the protective role (*HR* 0.75, 95% *CI* 0.43–1.29, *p* = 0.29), which was not present in those with SPPB value < 8 (*HR* 0.94, 95% *CI* 0.68–1.29, *p* = 0.71). Using individual participant-data metanalysis, results were consistent with the aggregated data analyses above reported (Fig. 4).

## Discussion

The major findings from the present analysis investigating the intricate relationship between sex, physical performance, and outcome in older ACS adults are:

i). Compared with men, women had a higher unadjusted rate of all-cause death
ii). Compared with men, women had a lower physical performance as assessed by SPPB
iii). After adjustment for potential confounding factors, SPPB values emerged as a strong predictor of all-cause death
iv). Physical performance and sex showed a significant interaction showing a greater protective effect of female sex for higher SPPB values

A recent study by DeFilippis et al. analyzed the difference in outcomes between men and women who experienced a myocardial infarction at young age [1]. All-cause death appeared to be significantly more frequent in young women when compared with men, and female sex was an independent predictor of outcome [1]. If the data are consistent also in older adults is still unknown. This concept is of paramount importance in the growing population of older adults and its projected to expand in the following years. Recent analyses about sex-based outcomes after PCI demonstrated that women had an increased 5-year risk of death, myocardial infarction, and revascularization compared with men [5]. However, these analyses did not involve just ACS, but the wide spectrum of coronary artery disease presentation. Furthermore, the analyzed population was collected from randomized clinical trials where older adults were underrepresented; therefore, the mean age of the study population was low (around 66 years vs. 81 years of the present analysis). A previous analysis of LONGEVO-SCA populations underlined that frail octogenarian women had a worse 6-month prognosis, introducing the potential effect of frailty on sex-related outcomes [20]. Our study is the first study investigating the long-term prognostic role of the intricate relationship between sex and physical performance in a large population of older ACS adults. Crude mortality is higher in women despite no higher rate of comorbidities. As a matter of fact, only a history of hypertension was more common in women. In contrast, other relevant comorbidities, such as diabetes, dyslipidemia, previous MI, history of ischemic heart disease, peripheral artery disease, and the evidence of multivessel coronary artery involvement, were more frequent in men. According to literature data, women resulted in being older and more compromised at presentation. The univariate analysis confirmed the adverse prognostic role of female sex, consistent with the literature, but after adjusting for confounding factors, sex showed a neutral effect in predicting all-cause death. This observation changes when we consider the physical performance as a potential effect modifier of the relationship between sex and mortality. We found that physical performance, as measured by SPPB, was a strong predictor of all-cause mortality, and its distribution between men and women significantly differed, being poor in the latter group. Thus, it is not surprising that after adjustment for SPPB value we observed a paradigm shift in the prognostic role of female sex. Not only did women not have a poor prognosis compared to men, but female sex was a protective factor in older ACS patients with preserved physical performance. These results could be explained considering the important emerging role of physical performance in outcome prediction: as previously reported, physical performance represents a new risk factor in older ACS patients [8, 9, 13, 14]. The present study demonstrated that this new risk factor overcame sex effect on outcome. These results have several meanings.

First, our study further highlights and reinforces the importance of physical performance assessment in studies involving older adults. Without this crucial parameter, there is the risk of underestimating the differences between groups and misunderstanding the role of a variable or the effectiveness of medical or interventional therapy. Second, our data suggest that the sex-related impact on mortality after MI is age-dependent. While female sex emerged as a negative prognostic marker in younger patients (≤65 years), it seems to be protective in those older and with preserved or mildly reduced physical performance (SPPB value ≥ 8 points). Third, sex is an unchangeable factor, whereas the physical performance status is not. Cardiac rehabilitation and exercise intervention are safe and effective in older ACS patients and can improve SPPB values [11, 12, 21]. Unfortunately, women tend to participate less in exercise programs [22]. This issue is even more striking considering that female sex may exert a protective effect in subjects with good physical performance and should motivate physicians to support the implementation of exercise programs in women after ACS.

### Study limitations

Our study suffers from some limitations. First, this is a not prespecified post hoc analysis from three different and independent studies performed for different primary aims. Thus, the present findings should be considered hypothesis-generating and should be confirmed by other independent larger studies. Second, physical performance was only assessed with SPPB. Therefore, we are not able to quantify the benefit of this scale compared to other tools in this large population. However, previous studies demonstrated that SPPB had a better prognostic role than other frailty tools in several populations, including older ACS adults [16, 17]. Third, although our dataset was extensive and complete, we may not exclude the presence of potential confounding factors not captured in our analysis. This is a crucial point, because older ACS patients are a peculiar population where several factors may play a role and may show a different weight as compared to that of younger populations. Fourth, the LONGEVO SCA study was focused on ACS patients without ST-segment elevation at hospital admission. Finally, the recruitment of patients was performed in the cardiology units of two countries (Italy and Spain). Therefore, our findings require further confirmation in a larger scenario.

### Conclusions

In a large real-life population of older ACS adults, after correction for clinical features and physical performance status (namely SPPB values), female sex was not related to adverse prognosis in terms of all-cause death. On the contrary, we found that female sex played a protective role in older ACS patients with preserved physical performance.

## Supplementary Information

**Additional file 1: Fig. S1.** Directed acyclic graph

**Additional file 2: Fig. S2.** Survival curves after adjustment for clinical and laboratory features.

**Additional file 3: Fig. S3.** Survival curves after adjustment for clinical and laboratory features and SPPB

## Data Availability

Datasets used and/or analyzed during this study are available from the corresponding author on reasonable request.

## Abbreviations

ACS: Acute coronary syndrome
DAG: Directed acyclic graph
FRASER: Frailty in Elderly Patients Receiving Cardiac Interventional Procedures
HULK: Physical Activity Intervention for Elderly Patients with Reduced Physical Performance after ACS
LONGEVO-SCA: Impacto de la Fragilidad y otros Síndromes Geriátricos en el Manejo y Pronóstico Vital del Anciano con Síndrome Coronario Agudo sin Elevación de Segmento ST
SPPB: Short Physical Performance Battery
