# Supplementary figures and images for: The impact of sex and physical performance on long-term mortality in older patients with myocardial infarction
Source: BMC Med. 2022 Jan 20;20:15. doi: 10.1186/s12916-021-02211-1 (PMC8772095; doi:10.1186/s12916-021-02211-1)

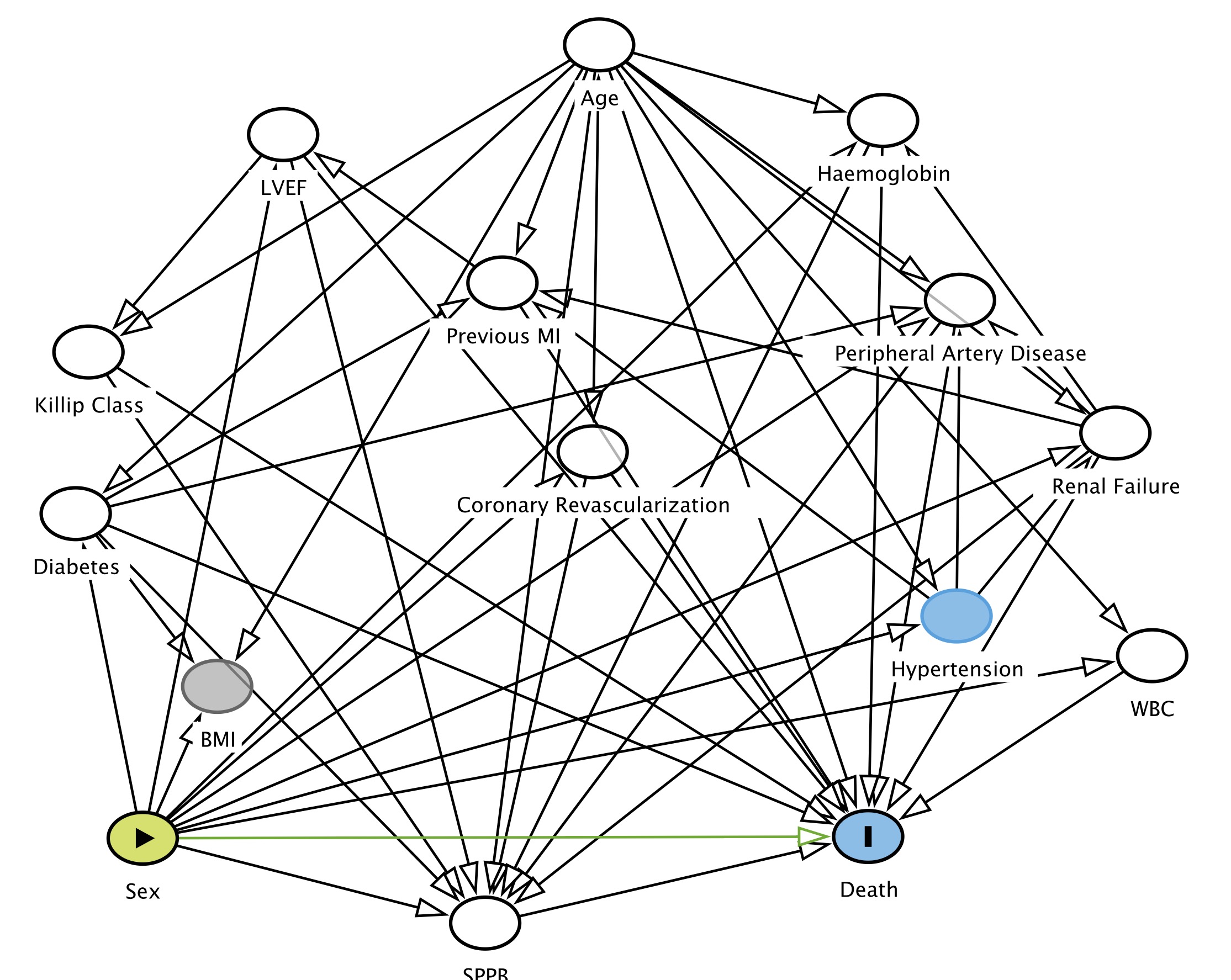

Supplement: Supplementary file 1 — Additional file 1: Fig. S1. Directed acyclic graph [file 12916_2021_2211_MOESM1_ESM.jpg]

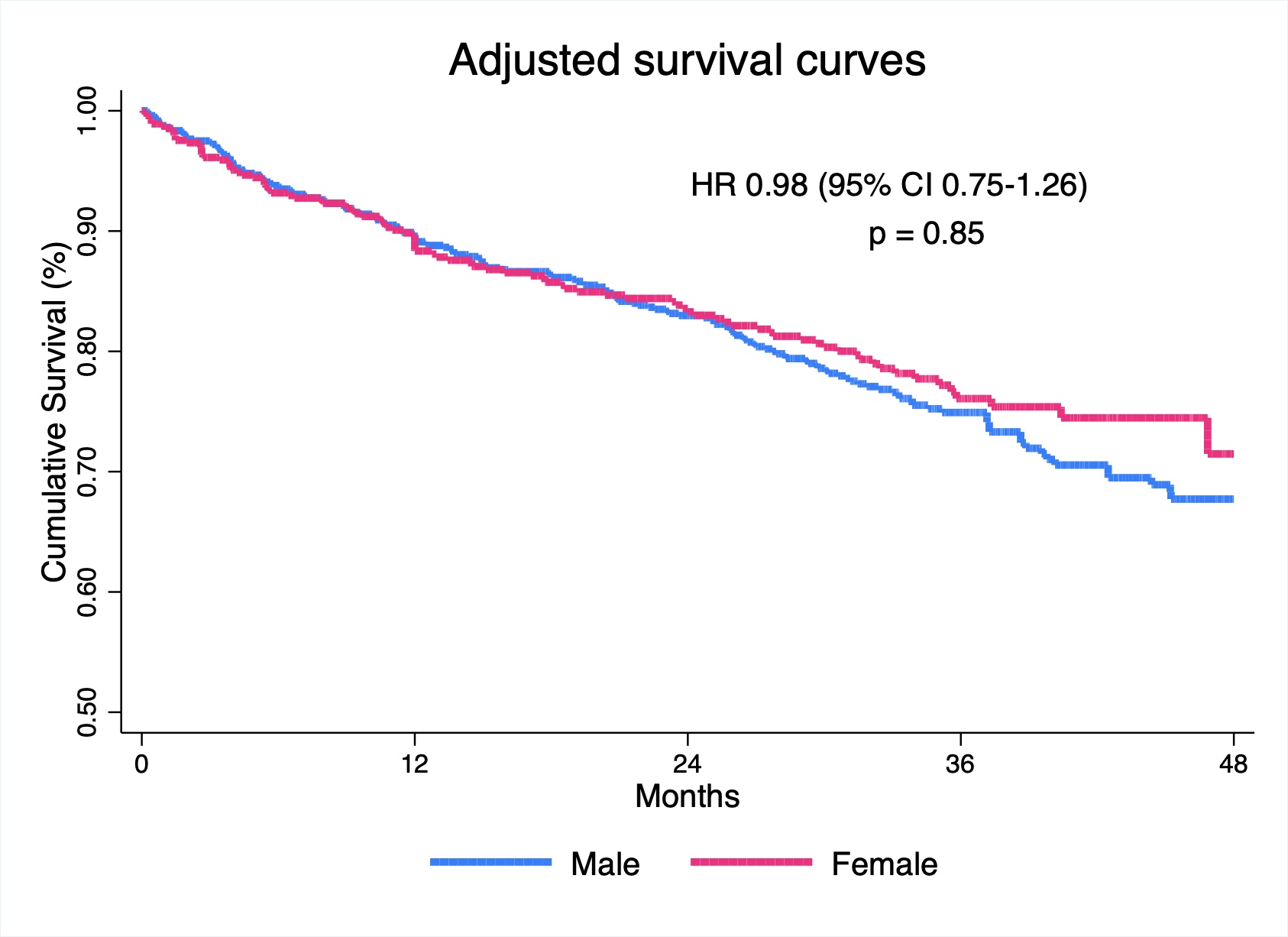

Supplement: Supplementary file 2 — Additional file 2: Fig. S2. Survival curves after adjustment for clinical and laboratory features. [file 12916_2021_2211_MOESM2_ESM.jpg]

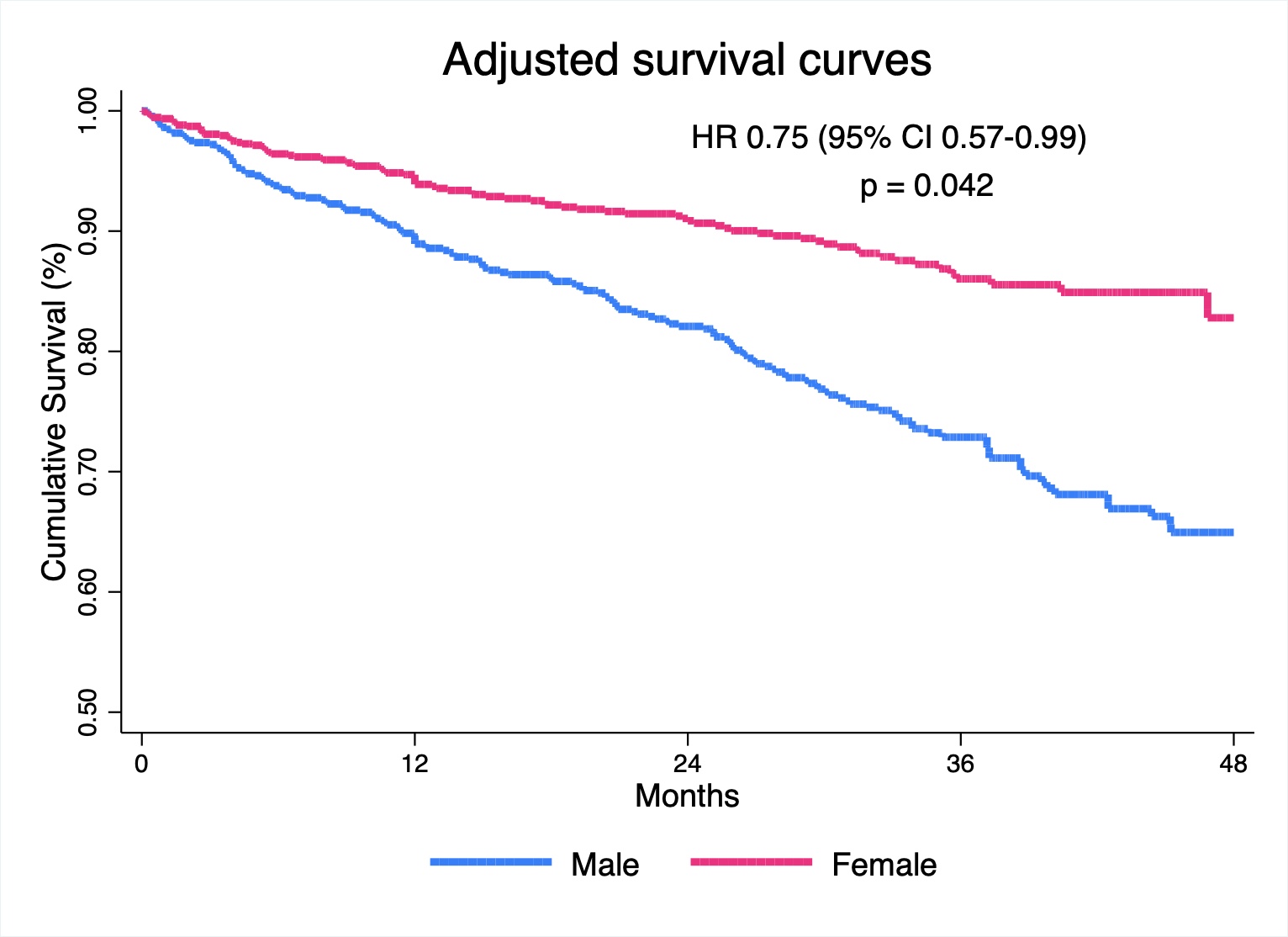

Supplement: Supplementary file 3 — Additional file 3: Fig. S3. Survival curves after adjustment for clinical and laboratory features and SPPB [file 12916_2021_2211_MOESM3_ESM.jpg]
